# Barriers to Colorectal Cancer Screening: Physician and General Population Perspectives, New Mexico, 2006

**Published:** 2011-02-15

**Authors:** Richard M. Hoffman, Robert L. Rhyne, Deborah L. Helitzer, S. Noell Stone, Andrew L. Sussman, Robyn Viera, Teddy D. Warner, Elizabeth E. Bruggeman

**Affiliations:** New Mexico VA Health Care System. Dr Hoffman is also affiliated with the Department of Medicine, University of New Mexico School of Medicine, Albuquerque, New Mexico; University of New Mexico School of Medicine, Albuquerque, New Mexico; University of New Mexico School of Medicine, Albuquerque, New Mexico; University of New Mexico School of Medicine, Albuquerque, New Mexico; University of New Mexico School of Medicine, Albuquerque, New Mexico; University of New Mexico School of Medicine, Albuquerque, New Mexico; University of New Mexico School of Medicine, Albuquerque, New Mexico; New Mexico Department of Health, Cancer Prevention and Control Section, Chronic Disease Prevention and Control Bureau, Albuquerque, New Mexico

## Abstract

**Introduction:**

Colorectal cancer (CRC) screening rates are low in New Mexico. We used statewide surveys of primary care physicians and the general population to characterize CRC screening practices and compare perceptions about screening barriers.

**Methods:**

In 2006, we surveyed 714 primary care physicians in New Mexico about their CRC screening practices, beliefs, and perceptions of patient, provider, and system barriers. A 2004 state-specific CRC screening module for the Behavioral Risk Factor Surveillance System (BRFSS) survey asked 3,355 participants aged 50 years or older why they had not ever or had not recently completed a fecal occult blood test (FOBT) or lower endoscopy.

**Results:**

The 216 physicians (30% response rate) reported offering screening to a median 80% of their average-risk patients in the past year and estimated that a median 50% were current with screening. They attributed low screening proportions mainly to patient factors (embarrassment, fear of pain, lack of insurance). However, just 51% of physician respondents used health maintenance flow sheets, and only 13% used electronic medical records to identify patients due for CRC screening. The BRFSS respondents most often reported that lack of physician discussion was responsible for not being current with screening (45% FOBT, 34% endoscopy); being asymptomatic was also often cited as an explanation for lack of screening (22% FOBT, 36% endoscopy).

**Conclusion:**

Physicians and adults in the general population had markedly different perspectives on barriers to CRC screening. Increasing screening may require system supports to help physicians readily identify patients due for CRC testing and interventions to educate patients about the rationale for screening.

## Introduction

Colorectal cancer (CRC) is the fourth most frequently diagnosed cancer in New Mexico and the second leading cause of cancer death (1). In 2009, an estimated 800 new CRC cases were diagnosed in New Mexico, and approximately 300 people died from this disease. Although CRC screening reduces both CRC incidence and mortality (2-4), just over half of New Mexican adults aged 50 years or older are considered current with screening (5). Consequently, fewer than half of the cancers in New Mexico are being diagnosed at an early stage, when they can be most effectively treated (6).

In New Mexico, the Clinical Prevention Initiative, a statewide partnership of health care organizations supported by the New Mexico Department of Health and the Centers for Disease Control and Prevention (CDC) (7), works to increase the delivery of high-impact preventive services (8) by targeting primary care providers. To guide interventions, the Clinical Prevention Initiative CRC workgroup decided to survey primary care providers to assess perceived barriers to CRC screening. The Department of Health had also recently collected population-based data on screening barriers through CDC's Behavioral Risk Factor Surveillance System (BRFSS) survey.

Although numerous barriers and facilitators to CRC screening have been identified in the literature (9-14), including patient, provider, health system, and policy factors, few surveys have been able to compare contemporaneous responses from providers and a general population (15). Furthermore, many studies were conducted before Medicare began reimbursing for screening colonoscopy in 2001 and before the 2002 publication of US Preventive Services Task Force (USPSTF) guidelines strongly recommending CRC screening (16), which substantially altered screening practices (5,17). Results from other states or regions may not necessarily be applicable in New Mexico; a lower proportion of New Mexicans is screened for CRC compared with national averages (54.8% vs 57.3%) (5). Additionally, national data show declining CRC incidence from 1997 through 2006 for both sexes and all major racial/ethnic groups (18); however, incidence in New Mexico has been stable or increasing among Hispanics and American Indians (6). New Mexico is the fifth largest state but only the 36th most populated state and has substantially higher than national population percentages of Hispanics (45% vs 15%) and American Indians (10% vs 1%) (19). Given New Mexico's anomalous trends in CRC incidence and diverse minority populations, the primary objective of our study was to comprehensively characterize provider-reported practices and barriers for CRC screening. A secondary objective was to compare these provider perceptions with those of the general population.

## Methods

### Physician survey

A team of content and methodology experts at the University of New Mexico Cancer Center developed the primary care physician survey in 2005. Some items were adapted from a National Cancer Institute survey on CRC screening barriers (15). We revised the survey after pilot testing it with a sample of 5 primary care physicians who assessed content and face validity. The final 34-item survey was divided into 6 sections that addressed the practice strategies and system support for performing screening (patient education, reminders, tracking), the rationale for recommending screening (influential factors), cancer screening beliefs and practices (perceived effectiveness, testing recommendations, patient adherence), screening barriers (patient, provider, system), practice characteristics (type, location, size), and physician characteristics (demographics, specialty). (Readers can obtain a copy of the survey from the corresponding author.) Screening practice items were based on USPSTF recommendations (16) for average-risk patients aged 50 years or older and included the options of colonoscopy, flexible sigmoidoscopy, barium enema, or fecal occult blood testing (FOBT). We used 6-point rating scales ranging from "never"/"not" = 1 to "most"/"very" = 6 to measure survey items assessing screening beliefs, practices, and barriers. The human research review committee of the University of New Mexico approved the physician survey.

We identified potential respondents from the 2006 New Mexico Board of Medical Examiners provider list, which contains the self-identified specialties and addresses for licensed physicians in New Mexico. The eligibility criterion was a primary specialty listing of internal medicine, family medicine, general practice, or geriatrics. We excluded physicians with secondary specialties that indicated that they were not primary care providers, including medical subspecialties, urgent care, or administration.

We mailed 964 surveys in June 2006 accompanied by a cover letter signed by leaders of primary care professional organizations and the New Mexico Department of Health. We subsequently mailed surveys approximately 3 and 5 months later to nonrespondents. We did not offer compensation for participating in the survey. On the basis of returned mailings, updated Medical Examiners lists, and survey responses, we excluded 123 physicians from our denominator because we could not locate them and another 127 physicians who either were no longer licensed in New Mexico (retired or moved) or who were not primary care providers. We were left with a denominator of 714 respondents and 216 completed surveys, for a response rate of 30%.

### Population-based survey

The study participants were a population-based sample of respondents to the 2004 New Mexico BRFSS, a federally funded annual survey implemented in collaboration with state health departments. Random-digit dialing methods were used to derive a probability sample of households with telephones to collect data on health-related behaviors and risk factors for respondents aged 18 years or older. In 2004, the New Mexico Department of Health designed a module that was administered to 6,390 adults aged 50 years or older that asked about CRC screening with a home FOBT and lower endoscopy. Overall, 3,355 participants who reported being not current or never tested with either home FOBT or lower endoscopy were asked to provide the most important reason for the lack of testing. These participants included 1,695 who had never performed a home FOBT and 798 who had not been tested in the past year, as well as 1,504 who never had undergone a sigmoidoscopy or colonoscopy and 297 who had not been tested in the past 5 years.

### Statistical analyses

For the physician survey, we used descriptive statistics to characterize providers, practices, screening practices, and ratings of barriers to screening. We conducted a series of exploratory factor analyses on the patient, provider, and system barriers assessed by the survey to confirm that survey items clustered within these 3 distinct groups of barriers. We used factorial within-subjects multivariate analyses of variance to directly compare physician rating levels within each set of barriers to CRC screening (20).

To account for the complex survey design of the BRFSS, we used Stata 9.0 (StataCorp LP, College Station, Texas) to estimate percentages and their 95% confidence intervals (CIs) by demographic characteristics. All estimates represent weighted population-based estimates for residents aged 50 years or older who responded to the 2004 New Mexico BRFSS.

## Results

### Physician survey

Among the physician respondents who completed these survey items, most were middle-aged white men who practiced in urban areas (Table 1). Almost half practiced in physician-owned, single-specialty practices, and most worked in small groups.

Few practices used an electronic medical record to identify patients due for CRC screening and most did not have a mechanism to ensure that patients completed FOBT tests (Table 2). However, few respondents found it difficult to arrange CRC screening.

The most influential factors for screening, on a 6-point scale from 1 (not at all influential) to 6 (very influential), were evidence from the medical literature and professional guidelines. Respondents considered screening colonoscopy to be the most effective strategy for reducing CRC mortality. They were less enthusiastic about FOBT, flexible sigmoidoscopy, and barium enema.

Colonoscopy was recommended at least some of the time for screening average-risk patients by 94% of respondents compared with 86% for FOBT. Overall, 74% of respondents never recommended flexible sigmoidoscopy, and 74% never recommended barium enema. Nearly all respondents reported that they began screening average-risk patients at age 50 for each of the screening procedures except FOBT, for which 23% reported that they began screening patients in their 40s. The mean (standard deviation) screening interval in years was 1.4 (1.6) for FOBT, 8.2 (2.6) for colonoscopy, 6.3 (2.4) for barium enema, and 4.5 (1.3) for flexible sigmoidoscopy. Nearly all respondents indicated that they had an upper age limit for screening with colonoscopy, barium enema, and flexible sigmoidoscopy; about half indicated that they would not screen beyond age 80. However, 63% of respondents reported not having an upper age limit for FOBT screening.

In the past year, respondents estimated offering CRC screening to a median (interquartile range) 80% (70%-95%) of their patients aged 50 years or older. Overall, respondents estimated that 50% (40%-70%) of their eligible patients were current with CRC screening recommendations.

Only 3 patient barriers were rated 4 or higher on the 6-point rating scale: embarrassment/anxiety, fear of pain, and inadequate insurance (Table 3). Physician barriers were all rated below the midpoint of 3.5 on the 6-point rating scale, indicating that physicians did not see themselves as often creating barriers to screening. Similarly, physicians generally did not perceive system factors, which were categorized into implementing screening and obtaining lower endoscopy, as creating barriers to screening.

### Population-based survey

Among the 3,355 respondents to the BRFSS module questions about lack of screening, 58% were aged 50 to 64 years, 47% were men, 67% were non-Hispanic whites, and 26% were Hispanic. By far, the most frequently cited response for never having undergone CRC screening or not being current with either FOBT or lower endoscopy was that a doctor never suggested testing (Table 4). A substantial proportion of participants indicated that lack of symptoms was the reason for not being screened. Few reported testing being distasteful or embarrassing, having fear of cancer, or having fear of pain with testing as barriers. Furthermore, access and cost issues were not frequently listed as barriers for FOBT, though they were slightly more common for endoscopic procedures.

## Discussion

We found that physicians and patients had markedly different perspectives on barriers to CRC screening. Although physicians reported offering screening to most of their average-risk patients, they acknowledged limited adherence. Physicians most often cited patient factors as being frequent barriers, including fear of pain, embarrassment and anxiety about testing, lack of insurance, and lack of knowledge about cancer and screening. Meanwhile, population-based data from adults aged 50 years or older showed that they rarely considered discomfort or embarrassment to be a primary barrier. Respondents were far more likely to report that lack of a physician recommendation or lack of symptoms prevented them from getting tested.

Other investigators have also noted a discrepancy between physician and patient perspectives on barriers, though not at a statewide level. Klabunde and colleagues compared results from nearly concurrent national surveys of providers (1999-2000 Survey of Colorectal Cancer Screening Practices) and adults in the general population (2000 National Health Interview Survey [NHIS]) (15). Investigators asked a nationally representative sample of primary care providers to rate the importance of patient and health care system barriers to screening with either FOBT or lower endoscopy. More than 90% of the physician respondents identified at least 1 major barrier, more often patient-related (80%) than system-related (68%). The most frequently cited patient barriers were embarrassment/anxiety about testing (56%), lack of awareness of screening/not perceiving the seriousness of CRC (48%), and fear of finding cancer (28%). The most frequently cited system barrier was financial (46%). In contrast, only 1% of NHIS respondents reported concerns about pain or embarrassment as major barriers for lower endoscopy. The most frequently cited barriers by patients were not seeing a need for testing/lack of awareness (51%), which was concordant with the physicians' responses; however, 21% also reported that their doctor did not order or recommend the test.

Physicians' failure to discuss screening is a common theme in patient surveys. Even though lack of time to discuss screening was not seen as a frequent barrier by our respondents, the literature suggests otherwise. Yarnall and colleagues estimated that it would take 7.4 hours a day for a provider in an adult primary care practice to address the preventive services deemed effective by USPSTF (21). Competing health demands can make it difficult to address screening during routine office visits, and a meta-analysis found that conducting a prevention visit was significantly associated with being able to deliver more screening (22).

Additionally, only one-third of our respondents reported that their practice had written policies for CRC screening, and availability of tracking systems and electronic medical records was limited. This may be an unrecognized provider barrier to discussing screening. Inadequate use of office systems has been identified as a major barrier for achieving screening (23,24). Developing office policies is seen as a necessary first step to ensuring system changes (25), and employing a tracking system can facilitate effective screening by identifying patients who are due for screening (or surveillance) — and ensuring that results of screening and diagnostic tests are documented (24).

Physician-patient communication about CRC screening may be less than ideal. Our results suggest that physicians may not be fully aware of patients' attitudes and values toward screening. Ling and colleagues studied attitudes toward CRC screening among physicians and patients at a single academic practice (26). Physicians markedly overestimated test discomfort as a barrier compared with patients and underestimated the importance of test accuracy for patients. Physicians may also not recognize the importance of helping patients make informed decisions for screening. CRC screening is a complex issue because multiple testing options are available, and various criteria are used to assess risks (27,28). These concepts can be difficult to convey, and the literature suggests that discussions often do not take place (29) or are inadequate (30,31). One study analyzed audiotaped clinic visits and found that although 40% of discussions provided patients with background information about screening, most did not address alternatives (74%) or pros and cons (83%), or elicit patient preferences (83%). Conversely, providing patients with a CRC screening decision aid that informed them about cancer risks and available effective tests was associated with a significant increase in completing screening tests (32).

The physician survey responses also revealed some implicit barriers to effective screening. Physicians believed that colonoscopy was more effective than FOBT and flexible sigmoidoscopy. This finding mirrors national survey results (33) and may be attributed to guidelines that rate colonoscopy as the optimal test (34). However, the objective evidence for screening effectiveness for FOBT is based on randomized controlled trials (2), whereas only case-control and observational data support the effectiveness of colonoscopy (35-37). Consequently, USPSTF gives an overall "A" rating to CRC screening, without recommending any specific tests (27). In New Mexico, capacity for colonoscopy is limited (38), suggesting that using alternative tests may be necessary to achieve higher screening rates.

Another issue was the potential for overscreening elderly patients. Among respondents to the questions about stopping screening, 63% did not indicate any upper age for stopping FOBT, while 55% of those who set an upper age for colonoscopy would continue recommending screening past age 80. USPSTF recommends that screening not be offered for patients aged 85 years or older and offered only after a risk-benefit discussion with patients aged 76 to 84 years because patients with limited life expectancy have little expected benefit from screening (27). Other provider surveys have also indicated insufficient consideration of patient age when making screening recommendations (39,40). Although the FOBT is inexpensive and safe, false-positive results are common and abnormal studies require diagnostic colonoscopy. Screening patients who are unlikely to receive any benefit is an inefficient use of resources.

### Limitations

Our study had some potential limitations. The overall response rate to the physician survey was low, creating a potential selection bias if respondents were not representative of the population of New Mexico primary care physicians. However, a recent New Mexico Health Policy Commission (NMHPC) report suggests that the demographics of our sample were consistent with statewide data on primary care physicians (41). In 2008, the NMHPC reported that 43% of primary care physicians were aged 55 or older (vs 39% in our sample), 56.9% were men (vs 68%), and 48% were in the county containing Albuquerque (vs 47%). We also know that our denominator of potentially eligible physicians was not accurate and that we likely underestimated our response rate. We relied on the Board of Medical Examiners physician listings, which do not consistently characterize specialty or training status, so we may have misclassified specialists and trainees as being primary care physicians. Contact information is updated only every 3 years, so we could not be certain that we had correct addresses. However, our results in terms of practice patterns, system support, and barriers are consistent with those of other surveys reporting higher response rates (15,33). We were also unable to verify provider responses regarding screening practices and adherence.

The BRFSS data are subject to selection and recall bias, although reports of physicians failing to recommend screening are supported by national surveys (15) as well as directly observed patient encounters (31). Social desirability bias may have caused respondents to minimize fear and embarrassment as screening barriers. Finally, our data are ecologic; physician and BRFSS respondents are not directly linked, which could result in differing perceptions of barriers, particularly related to access.

### Conclusion

Our results suggest that CRC screening in New Mexico could be facilitated by information systems that readily identify patients who are due for screening and track test results. Physicians may also increase screening by educating patients about cancer and the rationale and options for screening. Physicians in a state with limited resources for cancer screening should also avoid potentially inefficient (not having an upper age limit for FOBT screening) and impractical (emphasizing screening colonoscopy) screening strategies.

## Figures and Tables

**Table 1:** Physician Demographic and Practice Characteristics, New Mexico Primary Care Physician Survey of Colorectal Cancer Screening Barriers (N = 216), 2006

**Characteristic**	Mean (SD) or %
**Age, y (n = 153)**	50.6 (9.9)
**Male sex (n = 152)**	68%
**Race (n = 121)**	
White	95%
Other	4%
**Hispanic or Latino ethnicity (n = 149)**	9%
**Years since graduating medical school (n = 153)**	23 (10.4)
**Specialty (n = 205)**	
Internal medicine	46%
Family medicine	46%
General practice	3%
Missing	5%
**Have a subspecialty (n = 216)**	11%
**Have a medical school faculty appointment (n = 155)**	50%
**Personally perform flexible sigmoidoscopy (n = 202)**	19%
**Proportion of outpatients aged ≥50 y (n = 189)**	71 (53.9)
**Practice site (n = 172)**	
Albuquerque (metro population >800,000)	47%
Other urban excluding Albuquerque (metro population >60,000)	15%
Small town or rural	38%
**Practice setting (n = 170)**	
Physician-owned	43%
Group or staff model health maintenance organization	13%
Community health center	13%
University	12%
Public health/Indian Health Service	8%
Private hospital or clinic	6%
Veterans Affairs/military	4%
**No. of physicians in practice setting (n = 172)**	
1	16%
2-5	47%
6-10	21%
≥11	15%
**Works in multispecialty practice (n = 175)**	45%
**No. of outpatients seen weekly (n = 175)**	73 (53.4)

Abbreviation: SD, standard deviation.

**Table 2:** Practice Strategies and System Support for Performing Colorectal Cancer Screening, New Mexico Primary Care Physician Survey of Colorectal Cancer Screening Barriers (N = 216), 2006

**Strategy/Support**	%
**How CRC screening education is provided (n = 216^a^)**	
Providers discuss screening	94
Staff discuss screening	21
Posters/brochures in the waiting room	19
Brochures handed to patients	12
Letters to patients	6
Electronic media	2
Other	1
None	1
**Practice has an electronic medical record (n = 216)**	44
**Ways provider identifies patients due for CRC screening (n = 216^a^)**	
Asking patient at clinic visit	66
Health maintenance flow sheet^b^	51
Progress note^c^	45
Electronic medical record	13
Do not identify	5
Chart tickler	3
Other	3
**Has method to track that patients complete and return FOBT tests (n = 208)**	
Yes	18
No	74
Does not order FOBT	8
**Has mechanism to ensure FOBT results are in the medical record (n = 193)**	80
**Has written policies or protocols for CRC screening (n = 205)**	33
**Patient notified about screening or diagnostic test results (n = 203)**	86
**Ways provider finds out about colorectal endoscopic results (n = 215^a^)**	
Mail	74
Medical record review	39
Electronic communication	16
Other	9
**Ways provider finds out about colorectal radiological procedure results (n = 207^a^)**	
Mail	76
Medical record review	38
Electronic communication	21
Telephone	6
Other	8
**Provider notifies patient about screening or diagnostic test results (n = 203)**	86
**Ways provider notifies patients about test results (n = 174^a^)**	
In person	63
Mail	50
Telephone	47
Electronic	5
**Perceived difficulty in arranging CRC screening (n = 202)**	
Not at all difficult or low difficulty	60
Moderately difficult	23
Very difficult	17

Abbreviations: CRC, colorectal cancer; FOBT, fecal occult blood test.

a Percentages do not total 100 because providers could select more than 1 response.

b Defined as a tracking system to remind health care providers when patients are due for various preventive services.

c Defined as documentation in the medical record of the issues addressed in a clinic visit.

**Table 3:** Physician Ratings of Patient, Provider, and System Barriers to Colorectal Cancer Screening, New Mexico Primary Care Physician Survey of Colorectal Cancer Screening Barriers (N = 216), 2006^a^

**Perceived Barrier**	Mean (SD)^b^
**Patient**	
Embarrassment or anxiety	4.7 (1.0)
Fear of pain	4.5 (1.1)
Inadequate insurance	4.0 (1.6)
Lack of knowledge about screening tests	3.8 (1.2)
Lack of knowledge about CRC risk	3.8 (1.2)
Lack of perceived CRC susceptibility	3.8 (1.2)
Lack of benefit for CRC screening	3.7 (1.2)
Logistical barriers	3.7 (1.2)
Low utilization of annual health maintenance visits	3.7 (1.2)
Fear of finding cancer	3.6 (1.2)
Competing demands	3.6 (1.2)
Lack of benefit for CRC treatment	3.4 (1.3)
Low literacy	3.2 (1.3)
Fatalism	3.2 (1.2)
Poor patient adherence	3.2 (1.3)
Cultural factors	3.2 (1.2)
Inability to perform preparation for procedures	3.1 (1.1)
Family or friends had bad CRC experience	2.9 (1.1)
Language (non- or weak English speaker)	2.7 (1.2)
Non-US citizenship status	2.5 (1.7)
Colonoscopy complications	2.3 (1.1)
Barium enema complications	1.7 (1.1)
Flexible sigmoidoscopy complications	1.6 (1.0)
**Provider**	
Limited accuracy of FOBT	3.1 (1.4)
Lack of time to discuss screening	2.5 (1.1)
Lack of sigmoidoscopy skills	2.1 (1.8)
Lack of time to arrange screening	2.0 (1.1)
Difficulty counseling about screening	1.8 (1.0)
Complexity of screening options	1.7 (1.0)
Questions about efficacy of screening	1.7 (1.0)
**System barriers: implementing screening**	
Lack of screening system reminder	2.8 (1.4)
Lack of support staff for follow-up	2.6 (1.5)
Lack of FOBT result tracking system	2.4 (1.3)
Lack of patient educational material	2.3 (1.3)
Inadequate reimbursement for screening	2.0 (1.4)
**System barriers: obtaining lower endoscopy**	
Long waits to get lower endoscopy	3.0 (1.5)
Lack of resources for screening procedures	2.6 (1.7)
Difficulty scheduling lower endoscopy	2.6 (1.4)
Lack of resources for diagnostic procedures	2.3 (1.5)
Poor feedback on procedural results	2.2 (1.2)

Abbreviations: SD, standard deviation; CRC, colorectal cancer; FOBT, fecal occult blood test.

a Barriers were rated by providers for perceived frequency using a 6-point scale from 1 = "never"/"not" to 6 = "most"/"very".

b A multivariate analysis of variance showed that means differing by ≥0.2 were significantly different at *P* < .05 by Fisher's least significant difference method for all barriers except for system barriers, for which means differing by ≥0.3 were significant.

**Table 4:** Reasons for Not Undergoing Colorectal Cancer Screening Reported by Adults Aged 50 Years or Older (N = 3,355) in New Mexico, Behavioral Risk Factor Surveillance System (BRFSS), 2004^a^

**Reason**	Never Had Home FOBT, % (95% CI), n = 1,695	No Home FOBT in Previous Year, % (95% CI), n = 798	Never Had Lower Endoscopy, % (95% CI), n = 1,504	No Lower Endoscopy in Previous 5 Years, % (95% CI), n = 297
Physician never suggested	36.6 (33.9-39.4)	45.3 (41.2-49.4)	35.8 (32.9-38.7)	33.9 (27.5-40.8)
Physician said not necessary	4.4 (3.3-5.7)	9.0 (6.8-11.9)	3.0 (2.1-4.3)	5.8 (3.4-9.6)
Did test in physician office	13.0 (11.3-15.0)	5.4 (3.9-7.6)	NA	NA
No symptoms	30.7 (28.2-33.4)	21.9 (18.8-25.2)	33.5 (30.7-36.4)	36.3 (30.1-43.0)
No family history	2.6 (1.8-3.9)	1.1 (0.5-2.5)	4.6 (3.5-6.0)	3.9 (1.7-9.0)
Cost too high or not covered	0.9 (0.5-1.4)	1.0 (0.5-2.0)	4.5 (3.4-6.0)	4.8 (2.9-8.0)
Too young	0.7 (0.3-1.7)	0	0.9 (0.5-1.7)	0
Too old	0.2 (0.1-0.7)	1 (0.4-3.7)	0.6 (0.2-1.6)	0
No time	1.6 (0.9-2.9)	2.7 (1.6-4.6)	2.6 (1.6-4.1)	1.3 (0.6-2.9)
Distasteful	2.2 (1.5-3.3)	1.4 (0.7-2.6)	4.0 (3.0-5.2)	4.0 (2.2-7.4)
Embarrassing	0.5 (0.3-0.9)	0.6 (0.1-2.3)	1.4 (0.7-2.6)	0.2 (0.0-1.5)
Fear of cancer	0.3 (0.1-0.9)	0	0.8 (0.4-1.5)	0
Test painful	NA	NA	2.3 (1.6-3.4)	4.0 (2.2-7.1)
Do not know where to get test	0.3 (0.1-0.6)	0.1 (0.0-1.0)	0.1 (0.0-0.9)	0.4 (0.1-2.6)
Do not know how to get test	0.8 (0.4-1.6)	0.1 (0.0-0.5)	0.1 (0.0-0.4)	0
Never have routine checkup	2.3 (1.6-3.4)	5.6 (4.0-7.9)	2.3 (1.6-3.3)	1.1 (0.3-4.0)
Some other reason	2.8 (2.0-3.9)	4.7 (3.2-6.8)	3.5 (2.5-4.8)	4.4 (2.2-8.6)

Abbreviations: FOBT, fecal occult blood test; CI, confidence interval; NA, not applicable.

a Values represent weighted population-based estimates for 2004 New Mexico BRFSS respondents aged 50 y or older.
